# EEG Microstate Alterations in Anhedonia Subtypes of Major Depressive Disorder and Their Modulation by Intermittent Theta‐Burst Stimulation

**DOI:** 10.1155/da/1982576

**Published:** 2026-09-21

**Authors:** Jiang Wu, Zhengzhi Deng, Xinyuan Cheng, Shaoqing Li, Nan Qiu, Lan Hu, Zhihong Chen, Dezhong Yao, Li Pu, Hongmei Yan

**Affiliations:** ^1^ The Clinical Hospital of Chengdu Brain Science Institute, Sichuan Institute for Brain Science and Brain-Inspired Intelligence, China-Cuba Belt and Road Joint Laboratory on Neurotechnology and Brain-apparatus communication, School of Life Science and Technology, University of Electronic Science and Technology of China, Chengdu 610054, China, uestc.edu.cn; ^2^ Yangtze Delta Region Institute (Huzhou), University of Electronic Science and Technology of China, Huzhou 313001, China, uestc.edu.cn

**Keywords:** anhedonia, EEG microstates, intermittent theta-burst stimulation, major depressive disorder, Snaith–Hamilton pleasure scale

## Abstract

**Background:**

Anhedonia is a core symptom of major depressive disorder (MDD) and is associated with functional impairment, treatment resistance, and relapse. Emerging evidence suggests that anhedonia severity reflects distinct neural alterations; however, current neuromodulation protocols have not yet accounted for this clinical heterogeneity. We investigated whether intermittent theta‐burst stimulation (iTBS) differentially modulates electroencephalography (EEG) microstate dynamics across anhedonia subtypes and aimed to identify electrophysiological signatures for treatment stratification.

**Method:**

Fifty MDD patients were stratified into high‐anhedonia (HA, *n* = 25) and low‐anhedonia (LA, *n* = 25) groups based on Snaith–Hamilton pleasure scale (SHAPS) scores, alongside 25 healthy controls (HCs). Clinical assessment and resting‐state EEG data were collected at baseline and after a 2‐week course of iTBS targeting the left dorsolateral prefrontal cortex (DLPFC). Microstate parameters and transition probabilities (TPs) were analyzed.

**Results:**

At baseline, both patient groups showed reductions in microstates, MS_A and MS_D relative to HCs. The HA group additionally exhibited MS_C deficits, whereas the LA group showed MS_B reductions. TPs were decreased in both groups, with broader deficits in the HA group. Following iTBS, most microstate parameters of LA group no longer differed significantly from those of HCs, whereas the HA group exhibited only partial changes, with persistent abnormalities in MS_B duration, MS_C coverage, and MS_D parameters. Correlation analyses further revealed that, in the HA group, greater anhedonia improvement was associated with increased MS_A duration and decreased MS_B duration.

**Conclusion:**

iTBS exerts subtype‐specific modulation of large‐scale network dynamics in MDD with anhedonia. The LA group demonstrates treatment‐associated resolution, whereas the HA group displays enduring integration deficits involving the dorsal attention network (DAN), sensory network (SN), and default mode network (DMN). Notably, in the HA group, clinical improvement correlates with SN reorganization, suggesting that microstate changes may serve as a treatment response marker. MS_D dynamics represent a candidate signature for subtype classification, supporting stratification‐guided neuromodulation.

## 1. Introduction

Major depressive disorder (MDD) is a leading cause of global morbidity and mortality [1]. Anhedonia—the loss of pleasure or interest in rewarding experiences—is among its most disabling core symptoms [2]. Present in up to 70% of patients, anhedonia is associated with greater illness severity, prolonged depressive episodes, functional impairment, and increased relapse risk [3]. Neurobiological models implicate dysfunction within corticostriatal reward and motivational circuits [4], which exert widespread effects on striatal and prefrontal systems. Clinically, anhedonia strongly predicts poor prognosis and adverse treatment outcomes, positioning it as a priority target for precision psychiatry.

An accurate phenotypic assessment is essential to address this clinical gap. The Snaith–Hamilton pleasure scale (SHAPS) is widely used to quantify anhedonia severity [5–7]. It assesses four domains—interest/entertainment, social interaction, sensory experience, and food/drink—on a 14‐ to 56‐point scale, with higher scores indicating greater severity. Scores exceeding 28 typically indicate clinically significant anhedonia [8–10]. In contrast, the Hamilton depression rating scale‐17 (HAMD‐17), despite being the most widely used measure of depressive severity, contains only a single, nonspecific item addressing anhedonia [11, 12]. Although SHAPS and HAMD‐17 scores typically correlate, reductions in overall depression severity do not necessarily translate to remission of anhedonia symptoms [13, 14]. Residual anhedonia—characterized by persistently elevated SHAPS scores despite overall symptomatic improvement—remains prevalent and clinically consequential [15], underscoring a critical limitation in conventional depression assessment.

This assessment limitation carries substantial clinical consequences. MDD patients with anhedonia exhibit distinct clinical profiles, including marked social withdrawal, functional impairment, mood reactivity, brooding, and diurnal mood variation [16]. Persistent anhedonia—defined as inadequate symptom reduction following standard treatment—serves as an independent predictor of relapse, chronic dysfunction, and increased suicidality [17, 18]. Large‐scale cohort studies demonstrate that fluctuations in anhedonia closely parallel changes in suicidal ideation (SI) severity [19] and that targeted reduction of anhedonia decreases suicide risk independently of other depressive symptoms [20, 21]. A meta‐analysis encompassing over 11,000 participants confirmed that multiple subtypes of anhedonia are significantly associated with suicidality across both clinical and community samples [22]. Collectively, these findings position anhedonia not merely as a symptom dimension but as a critical prognostic marker and therapeutic target in MDD.

Repetitive transcranial magnetic stimulation (rTMS), including intermittent theta‐burst stimulation (iTBS), is a noninvasive neuromodulation strategy with established antidepressant efficacy, exerting its effects through the modulation of neurotransmitter systems, cerebral blood flow, and corticocortical/corticosubcortical connectivity [23, 24]. However, its therapeutic impact on anhedonia remains inconsistent. Naturalistic studies report substantial anhedonia improvement in treatment‐resistant depression, suggesting that baseline severity does not dictate response [25, 26]. Conversely, randomized controlled trials indicate that connectivity‐guided rTMS targeting the left dorsolateral prefrontal cortex (DLPFC)—a key cortical node regulating subcortical reward circuits—yields greater anhedonia reduction than sham stimulation [27]. Yet, other studies employing similar targeting protocols report minimal or nonsignificant effects [28, 29]. These discrepancies highlight two unresolved questions: (1) whether baseline anhedonia phenotypes stratify rTMS responsiveness and (2) which neural mechanisms underlie heterogeneous treatment outcomes.

Electroencephalography (EEG) microstate analysis offers a promising framework to address these questions [30, 31]. Microstates represent brief, quasi‐stable configurations of the scalp electric field (~80–120 ms) that serve as the fundamental building blocks of resting‐state brain dynamics [32]. Four canonical microstates (MS_A–D) have been identified. Source localization and multimodal imaging studies tentatively map these microstates onto distinct large‐scale networks: MS_A to the auditory network (AN), MS_B to the visual network (VN), MS_C to the default mode network (DMN), and MS_D to the dorsal attention network (DAN) [33]. Although these network assignments are derived from group‐level inverse solutions and remain probabilistic, they provide a validated heuristic for linking millisecond‐scale EEG dynamics to canonical brain networks.

Alterations in these microstates have been observed in MDD [34, 35], but their relationship to core, treatment‐resistant symptoms such as anhedonia remains unclear. One prior study examined microstates dynamics in the context of rTMS for anhedonia, reporting that increased MS_C predicted symptom improvement [36]. This preliminary finding suggests that microstate parameters may serve as tractable electrophysiological signatures of treatment response. Therefore, how rTMS modulates the microstate dynamics of different anhedonia subtypes is worthy of systematic investigation. In the present study, we investigated whether baseline anhedonia severity stratifies MDD patients into neurophysiologically distinct subtypes and whether these subtypes exhibit differential modulation following iTBS. Patients were classified into high‐anhedonia (HA) and low‐anhedonia (LA) groups based on SHAPS scores and compared with healthy controls (HCs). Resting‐state EEG was employed to quantify microstate parameters (duration, occurrence, and coverage) and transition probabilities (TPs). Our specific aims were to: (1) characterize baseline microstate alterations in the HA and the LA groups relative to HCs; (2) determine whether iTBS differentially modulates these abnormalities across subtypes; and (3) identify network‐ and subtype‐specific signatures—particularly within the DAN, sensory networks (auditory and visual network; SN), and DMN—that may inform treatment stratification. By linking anhedonia subtypes to neurophysiological dynamics and their modulation by iTBS, this work seeks to establish EEG microstate metrics as candidate electrophysiological signatures to guide subtype‐guided neuromodulation in MDD.

## 2. Methods

### 2.1. Participants

This study protocol was approved by the Ethics Committee of Chengdu Fourth People’s Hospital (Sichuan Province, China) and registered at ClinicalTrials.gov (NCT06417437). Written informed consent was obtained from all participants prior to enrollment. Recruitment was conducted in the psychiatric outpatient department between February 1, 2024, and January 20, 2025. Fifty right‐handed patients aged 18–60 years who met the Diagnostic and Statistical Manual of Mental Disorders, Fifth Edition (DSM‐V) [37] criteria for MDD and presented with clinically significant anhedonia were enrolled. Patients were stratified into two subgroups based on SHAPS scores: LA (28 < SHAPS ≤ 33, *n* = 25) and HA (SHAPS > 33, *n* = 25) groups. The cut‐off was selected for two reasons: (1) a score of 33 has been commonly used as the threshold for clinically significant anhedonia in MDD populations [38, 39]; and (2) three senior psychiatrists with clinical experience in using the SHAPS among Chinese MDD patients reached a consensus. All patients demonstrated preserved cognitive function. Additionally, 25 age‐, sex‐, and education‐matched HCs were recruited.

Inclusion criteria were (1) DSM‐V diagnosis of MDD; (2) Han ethnicity, age ≥18 years; (3) junior high school education level or above; (4) right‐handedness; (5) HAMD‐17 score ≥17; (6) no psychiatric medication use within the past 3 months, or on a stable regimen for at least 3 months with antidepressant use limited to selective serotonin reuptake inhibitors (SSRIs) and norepinephrine reuptake inhibitors (SNRIs); and (7) ability and willingness to participate, undergo treatment, and provide informed consent. Exclusion criteria comprised: (1) current or past organic brain diseases, major brain trauma, or personal/family history of epilepsy; (2) severe cardiovascular, hepatic, or renal disease; (3) severe physical illness; (4) substance use disorder (including alcohol or illicit drugs); (5) comorbid psychiatric diagnoses other than MDD; (6) pregnancy or lactation; (7) electroconvulsive therapy (ECT), rTMS, or other neuromodulatory intervention within the past 6 months; (8) implanted nerve stimulators or electronic/mental devices (e.g., pacemakers, defibrillators, stents, orthopedic implants); and (9) severe uncorrected visual or auditory impairments precluding neuropsychological testing.

### 2.2. TMS Therapy Course

All patients received twice‐daily iTBS sessions on weekdays, separated by a 1‐h interval. Stimulation was delivered using an iron‐core figure‐8 coil attached to a TMS device (Wuhan Yiruide Company, China). The left DLPFC target was localized using the international 10–20 EEG system (F3 electrode position) and verified via anatomical landmarks (5 cm anterior to the motor cortex hand area) [40, 41]. The stimulation parameters included bursts of 10 pulses, each made up of 3 pulses at 50 Hz, repeated every 200 ms (corresponding to a 5‐Hz theta frequency), generating 2 s of stimulation followed by an 8‐second rest. This cycle was repeated 60 times for a total of 1800 pulses. Individual resting motor threshold (RMT) values were determined 24 h prior to the first session using standard stimulation of thumb abductor muscle.

### 2.3. EEG Data Acquisition and Pre‐Processing

Resting‐state EEG was recorded while participants sat quietly with eyes closed to minimize ocular and myogenic artifacts [42]. Five minutes of EEG data were collected using a 64‐channel BrainAmp MR32 amplifier (Brain Products GmbH, Gilching, Germany) at a 1000 Hz sampling rate, with online reference at FCz and electrode‐skin impedance maintained below 10 kΩ. The cap layout followed the 10–20 system.

Offline preprocessing was conducted using EEGLAB software (2023.0 version). Signals were band‐pass filtered at 0.1–40 Hz, and ocular artifacts were removed using independent component analysis (ICA) [43]. Remaining artifacts were eliminated via expert visual inspection. Cleaned data were re‐referenced to the average reference [34], filtered at 2–20 Hz, and segmented into nonoverlapping 2‐second epochs. The first and last 30 s of data were discarded, and 100 artifact‐free epochs per participant were selected for subsequent microstate analysis. The number of artifact‐free epochs available per participant is detailed in Supporting information 1: Table S4.

### 2.4. Microstate Analysis

EEG microstates were analyzed using Cartool software [44]. For each EEG recording, global field power (GFP)—the spatial standard deviation of EEG potentials across electrodes—was computed at each time point. GFP peaks representing optimal quasi‐stable voltage topographies [45] were identified and clustered using an adapted k‐means algorithm [46] (*k* = 1–15; polarity ignored; Euclidean distance metric). The optimal number of clusters was determined via a meta‐criterion integrating seven optimization metrics [33]. Subject‐level centroids were then clustered again to generate group‐level microstate maps, from which the global templates used for subsequent fitting were derived. The resulting four microstate classes (MS_A–MS_D) were sorted and labeled according to the template ordering described by Koenig et al. [47], which provides normative reference topographies derived from a large healthy cohort, to ensure compatibility with the established microstate literature.

Each EEG time point was assigned to the microstate with the highest spatial correlation to the global templates. Temporal smoothing (12 ms half‐window), a Besag factor of 10, and exclusion of segments shorter than 20 ms [48] were applied to reduce noise. Parameters computed for each subject included duration (mean length of each microstate occurrence, in ms), occurrence (number per second), coverage (percentage of total recording time), and TPs between microstate classes. First‐order Markov‐chain models [49, 50] were used to compute both expected and observed TPs, where the latter represented actual transitions during the EEG sequence. The goodness‐of‐fit of the four‐class microstate model was evaluated by calculating the global explained variance (GEV) for each participant, defined as the proportion of total EEG signal variance explained by the fitted microstate model [33].

### 2.5. Statistical Analyses

Statistical analyses were performed using GraphPad Prism 9. Demographic variables were compared using chi‐square tests and one‐way analysis of variance (ANOVA). For clinical and microstate parameters, independent‐sample *t*‐tests were used for between‐group comparisons and paired‐sample *t*‐tests for pre–post changes, with Bonferroni correction applied. Two‐way repeated‐measures ANOVA (Group × Time) was performed to assess differential treatment responses between the LA and the HA groups, with partial *η^2^* reported as effect size. For TPs, false discovery rate (FDR) correction (Benjamini–Hochberg, *q* = 0.05) was applied separately to each family of comparisons (baseline, post‐intervention, and pre–post changes). GEV values were compared among groups using one‐way ANOVA and across sessions using paired *t*‐tests. Spearman correlations were performed separately for LA and HA groups to examine associations between clinical improvement (ΔSHAPS, ΔHAMD‐17, and ΔHAMA‐14) and changes in microstate parameters, with FDR correction within each group. Effect sizes are reported as *Cohen’s d* for *t*‐tests and partial *η^2^* for ANOVA.

## 3. Result

### 3.1. Clinical Outcome

Demographic and clinical characteristics of the LA, HA, and HC groups are summarized in Table 1. The three groups did not differ significantly in sex distribution (*χ^2^* = 0.118, *p* = 0.943, *η^2^* = 0.002), age (*F*(*2*, *72*) = 1.012, *p* = 0.369, *η^2^* = 0.027), or years of education (*F*(*2*, *72*) = 3.053, *p* = 0.078, *η^2^* = 0.076). As detailed in Supporting Information 1: Table S1, the LA and HA groups were well‐matched in illness duration, episode status (first‐episode vs. recurrent), and medication status (all *p* > 0.05). Following the 2‐week iTBS course, both patient groups exhibited significant reductions in depression and anxiety symptoms. HAMD‐17 scores decreased in the LA group (*t* = 21.650, *p* < 0.001, *Cohen’s d* = 4.330) and the HA group (*t* = 18.640, *p* < 0.001, *Cohen’s d* = 3.728). HAMA‐14 scores similarly declined in both groups (LA: *t* = 13.140, *p* < 0.001, *Cohen’s d* = 2.598; HA: *t* = 11.380, *p* < 0.001, *Cohen’s d* = 2.276). All patients achieved scores below clinical thresholds post‐treatment (HAMD‐17 < 8; HAMA‐14 < 7).

**Table 1:** Demographic and clinical characteristics of LA, HA, and HC groups.

Variable	LA group (mean ± SD)	HA group (mean ± SD)	HC group (mean ± SD
Demographic data			
Sex (Male/Female)	9/16	8/17	9/16
Age (years)	33.08 ± 9.90	35.24 ± 11.31	31.04 ± 8.23
Education (years)	13.80 ± 2.90	14.28 ± 2.68	15.44 ± 1.10
Clinical assessments			
SHAPS (pre)	30.20 ± 2.23	38.40 ± 3.48	23.44 ± 1.33
SHAPS (post)	24.80 ± 3.07	33.04 ± 4.72	—
HAMD‐17 (pre)	21.96 ± 3.77	22.32 ± 4.51	3.48 ± 1.48
HAMD‐17 (post)	4.96 ± 2.44	5.24 ± 1.75	—
HAMA‐14 (pre)	16.96 ± 4.92	16.44 ± 5.94	2.00 ± 0.81
HAMA‐14 (post)	4.52 ± 2.42	5.08 ± 1.87	—

*Note:* Values are presented as mean ± standard deviation (SD) unless otherwise indicated.

Abbreviations: HA, high anhedonia; HAMA‐14, Hamilton anxiety rating scale; HAMD‐17, Hamilton depression scale‐17; HC, healthy control; LA, low anhedonia; SHAPS, Snaith–Hamilton pleasure scale.

Crucially, anhedonia recovery differed markedly between the subtypes. In the LA group, SHAPS scores fell below the clinical threshold (*t* = 8.332, *p* < 0.001, *Cohen’s d* = 1.666) and no longer differed from HCs. In contrast, although the HA group also showed a significant reduction in SHAPS scores (*t* = 5.588, *p* < 0.001, *Cohen’s d* = 1.118), residual anhedonia persisted: post‐intervention SHAPS scores remained above the clinical threshold and were significantly higher than those of both the LA (*t* = 7.813, *p* < 0.001, *Cohen’s d* = 2.210) and the HC groups (*t* = 10.620, *p* < 0.001, *Cohen’s d* = 3.134).

### 3.2. Canonical Microstate Topographies

**Figure 1:**
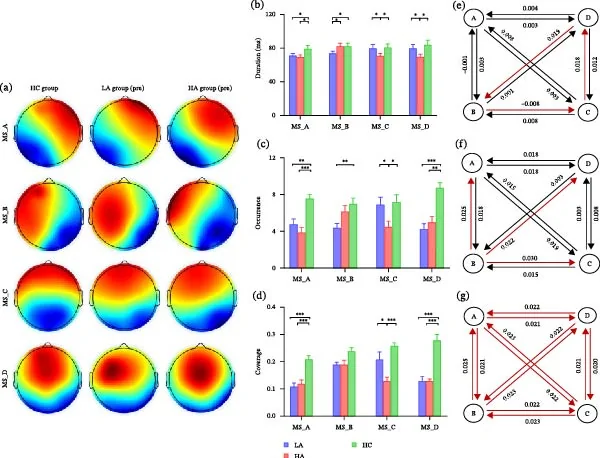
Baseline resting‐state microstate differences among LA, HA, and HC groups. Subpanels show: (a) microstate topographies; (b) microstate duration; (c) microstate occurrence; (d) microstate coverage; (e) TPs in the LA and HA groups; (f) TPs in the HC and LA groups; (g) TPs in the HC and HA groups. Red arrows indicate significant differences ( ^∗^ *p* < 0.05;  ^∗∗^ *p* < 0.01;  ^∗∗∗^ *p* < 0.001). All reported *p*‐values for transition probabilities are FDR‐corrected for multiple comparisons (*q* < 0.05). Effect sizes (*Cohen’s d*) are reported in the main text and Supporting Information 1: Table S2.

**Figure 2:**
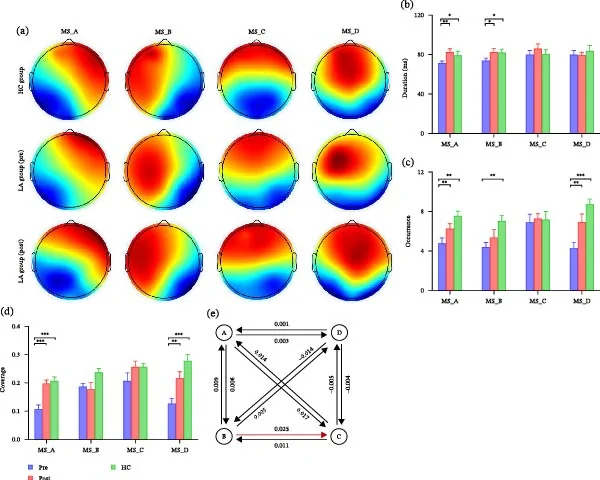
Microstate changes in the LA group pre‐ vs. post‐iTBS relative to HCs. Subpanels show: (a) microstate topographies; (b) microstate duration; (c) microstate occurrence; (d) microstate coverage; (e) TPs. Red arrow indicates significant differences ( ^∗^ *p* < 0.05;  ^∗∗^ *p* < 0.01;  ^∗∗∗^ *p* < 0.001). All reported *p*‐values for transition probabilities are FDR‐corrected for multiple comparisons (*q* < 0.05). Effect sizes (*Cohen’s d*) are reported in the main text and Supporting Information 1: Table S2.

**Figure 3:**
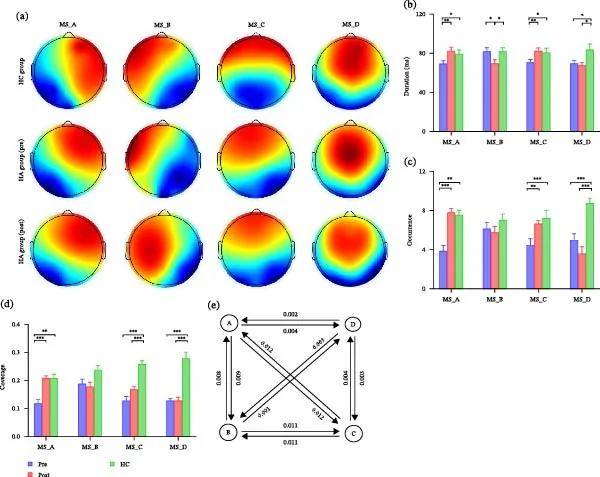
Microstate changes in the HA group pre‐ vs. post‐iTBS relative to HCs. Subpanels show: (a) microstate topographies; (b) microstate duration; (c) microstate occurrence; (d) microstate coverage; (e) TPs. Arrows indicate significant differences ( ^∗^ *p* < 0.05;  ^∗∗^ *p* < 0.01;  ^∗∗∗^ *p* < 0.001). All reported *p*‐values for transition probabilities are FDR‐corrected for multiple comparisons (*q* < 0.05). Effect sizes (*Cohen’s d*) are reported in the main text and Supporting Information 1: Table S2.

**Figure 4:**
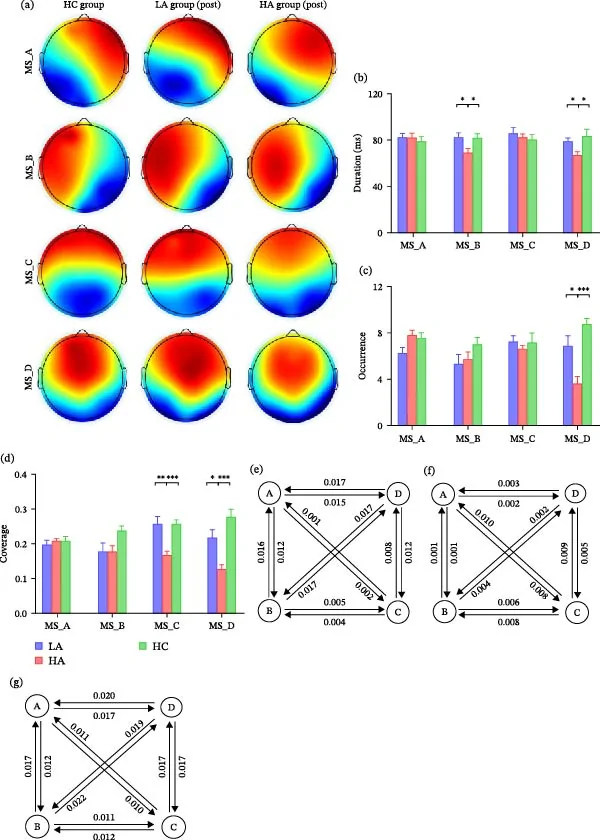
Post‐intervention resting‐state microstate differences among LA, HA, and HC groups. Subpanels show: (a) microstate topographies; (b) microstate duration; (c) microstate occurrence; (d) microstate coverage; (e) TPs in the LA and HA groups; (f) TPs in the HC and LA groups; (g) TPs in the HC and HA groups. Arrows indicate significant differences ( ^∗^ *p* < 0.05;  ^∗∗^ *p* < 0.01;  ^∗∗∗^ *p* < 0.001). All reported *p*‐values for transition probabilities are FDR‐corrected for multiple comparisons (*q* < 0.05). Effect sizes (*Cohen’s d*) are reported in the main text and Supporting Information 1: Table S2.

Global clustering analysis identified four canonical microstates (MS_A–D) across all groups and time points (Figures 1–4a). Consistent with established literature [33], these spatial topographies were characterized: left posterior–right anterior (MS_A), right posterior–left anterior (MS_B), anterior–posterior (MS_C), and fronto–central maximum (MS_D). The four‐class microstate model yielded comparable GEV values across groups and sessions, with no significant differences among groups at either baseline or post‐intervention or between pre‐ and post‐intervention within either patient group (Supporting Information 1: Table S6).

### 3.3. Resting‐State Microstates Differences Among HC, LA, and HA Groups at Baseline

At baseline, the HA group exhibited more widespread microstate abnormalities than the LA group. Both patient groups shared deficits in MS_A and MS_D. Specifically, MS_A duration was shorter in both the LA (*t* = 2.075, *p* = 0.048, *Cohen’s d* = 0.559) and the HA (*t* = 2.246, *p* = 0.030, *Cohen’s d* = 0.649) groups compared to HCs, with no significant difference between patient groups. MS_A occurrence and coverage were also reduced in both patient groups relative to HCs (occurrence: all *p* < 0.01; coverage: HC vs. LA: *t* = 3.898, *p* < 0.001, *Cohen’s d* = 1.591; HC vs. HA: *t* = 3.595, *p* = 0.002, *Cohen’s d* = 1.468). For MS_D, both patient groups showed reduced occurrence and coverage compared to HCs (occurrence: all *p* < 0.01; coverage: HCs vs. LA: *t* = 4.461, *p* < 0.001, *Cohen’s d* = 1.922; HCs vs. HA: *t* = 4.266, *p* < 0.001, *Cohen’s d* = 1.823). However, MS_D duration was significantly longer in the LA and HC groups than in the HA group (all *p* < 0.05), with no difference between the LA and the HC groups (Figure 1b).

Beyond these shared deficits, the LA group showed selective MS_B abnormalities. MS_B occurrence was reduced in the LA group compared to HCs (*t* = 3.146, *p* = 0.004, *Cohen’s d* = 1.189). MS_B duration was longer in the HA and HC groups than in the LA group (HCs vs. LA: *t* = 2.128, *p* = 0.039, *Cohen’s d* = 0.660; HA vs. LA: *t* = 2.095, *p* = 0.042, *Cohen’s d* = 0.611), with no difference between the HA and the HC groups. Conversely, the HA group showed additional MS_C deficits. Both LA and HC groups showed longer MS_C duration than the HA group (all *p* < 0.05), with no differences between the LA and the HC group (Figure 1b). MS_C occurrence was higher in LA and HC groups than in the HA group (all *p* < 0.05; Figure 1c). MS_C coverage was significantly greater in the LA and HC groups relative to the HA group (LA vs. HA: *t* = 2.070, *p* = 0.049, *Cohen’s d* = 0.812; HC vs. HA: *t* = 5.695, *p* < 0.001, *Cohen’s d* = 2.234). MS_B coverage did not differ significantly among groups (Figure 1d).

Regarding TPs, the HA group exhibited more pervasive network‐switching disruptions. Significant group differences survived FDR correction (Supporting Information 1: Table S2 for full results). The LA group showed higher TPs from MS_C to MS_D (*t* = 2.740, *p* = 0.026, *Cohen’s d* = 0.825) and from MS_D to MS_B (*t* = 3.389, *p* = 0.009, *Cohen’s d* = 1.020) compared to the HA group. Conversely, the LA group exhibited lower TPs from MS_B to MS_C than the HA group (*t* = 3.235, *p* = 0.018, *Cohen’s d* = 0.915; Figure 1e). The HC group displayed significantly higher overall TPs than the HA group (*p* < 0.05, FDR‐corrected; Figure 1f). Additionally, the HC group showed higher TPs than the LA group for MS_B to MS_A (*t* = 3.027, *p* = 0.020, *Cohen’s d* = 0.874), MS_B to MS_C (*t* = 3.989, *p* = 0.004, *Cohen’s d* = 1.152), and MS_B to MS_D (*t* = 2.655, *p* = 0.037, *Cohen’s d* = 0.767; Figure 1g).

### 3.4. Resting‐State Microstates Difference Between the LA Group (Pre‐ vs. Post‐Intervention) and the HC Group

Following iTBS, most previously altered microstate parameters in the LA group no longer differed significantly from HCs. MS_A (*t* = 3.112, *p* = 0.005, *Cohen’s d* = 0.635) and MS_B (*t* = 2.020, *p* = 0.048, *Cohen’s d* = 0.449) durations increased to levels comparable to HCs, whereas MS_C and MS_D durations remained unchanged (Figure 2b). MS_A (*t* = 3.139, *p* = 0.009, *Cohen’s d* = 0.906) and MS_D (*t* = 3.026, *p* = 0.001, *Cohen’s d* = 0.781) occurrence increased and were no longer different from HCs. MS_B occurrence, initially reduced relative to HCs (*t* = 3.146, *p* = 0.004, *Cohen’s d* = 1.189), was fully restored. MS_C occurrence remained unchanged (Figure 2c). MS_A (*t* = 7.654, *p* < 0.001, *Cohen’s d* = 2.210) and MS_D (*t* = 3.307, *p* = 0.006, *Cohen’s d* = 0.884) coverage increased, eliminating baseline differences, whereas MS_B and MS_C coverage showed no significant changes (Figure 2d). Regarding TPs, a significant increase was observed for the MS_B to MS_C transition (*t* = 3.760, *p* = 0.016, FDR‐corrected, *Cohen’s d* = 0.784), with no other transitions changing significantly (Figure 2e).

### 3.5. Resting‐State Microstates Difference Between the HA Group (Pre‐ vs. Post‐Intervention) and the HC Group

In the HA group, despite partial improvements after iTBS, several abnormalities persisted. MS_A (*t* = 3.047, *p* = 0.006, *Cohen’s d* = 0.635) and MS_C (*t* = 3.649, *p* = 0.001, *Cohen’s d* = 0.761) durations increased to HCs levels. By contrast, MS_B duration decreased (*t* = 2.596, *p* = 0.016, *Cohen’s d* = 0.541) and remained significantly lower than HCs (*t* = 2.470, *p* = 0.017, *Cohen’s d* = 0.714), despite the absence of a baseline difference. MS_D duration did not change and remained lower than HCs at both baseline (*t* = 2.392, *p* = 0.021, *Cohen’s d* = 0.691) and post‐intervention (*t* = 2.787, *p* = 0.018, *Cohen’s d* = 0.805; Figure 3b). MS_A (*t* = 6.092, *p* < 0.001, *Cohen’s d* = 1.523) and MS_C (*t* = 3.273, *p* = 0.006, *Cohen’s d* = 0.875) occurrence increased to HCs levels, while MS_B remained stable. MS_D occurrence did not change and remained lower than HCs at baseline (*t* = 4.070, *p* < 0.001, *Cohen’s d* = 1.616) and post‐intervention (HC: *t* = 5.462, *p* < 0.001, *Cohen’s d* = 2.168; Figure 3c). MS_A coverage increased to levels comparable to HCs (*t* = 6.610, *p* < 0.001, *Cohen’s d* = 1.908). However, MS_C (baseline: *t* = 4.947, *p* < 0.001, *Cohen’s d* = 1.940; post: *t* = 5.695, *p* < 0.001) and MS_D (baseline: *t* = 4.266, *p* < 0.001, *Cohen’s d* = 1.832; post: *t* = 4.069, *p* < 0.001) coverage remained significantly lower than HCs. MS_B coverage did not change (Figure 3d). No significant post‐intervention changes were observed in TPs (Figure 3e).

### 3.6. Resting‐State Microstate Differences Among HC, LA, and HA Group at Post‐Intervention

Post‐treatment, the HA group retained abnormalities across multiple microstate parameters, whereas the LA group no longer differed from HCs on any measure. For duration, MS_B (LA vs. HA: *t* = 2.764, *p* = 0.011, *Cohen’s d* = 0.878; HC vs. HA: *t* = 2.470, *p* = 0.017, *Cohen’s d* = 0.714) and MS_D (LA vs. HA: *t* = 2.820, *p* = 0.015, *Cohen’s d* = 0.853; HC vs. HA: *t* = 2.787, *p* = 0.018, *Cohen’s d* = 0.805) were significantly longer in the LA and HC groups than in the HA group, with no difference between the LA and HC groups. MS_A and MS_C durations did not differ among groups (Figure 4b). For occurrence, MS_D occurrence was higher in the LA and HC groups than in the HA group (LA vs. HA: *t* = 2.469, *p* = 0.021, *Cohen’s d* = 0.980; HC vs. HA: *t* = 4.069, *p* < 0.001, *Cohen’s d* = 2.168), with no difference between the LA and the HC groups. MS_A, MS_B, and MS_C occurrence did not differ among groups (Figure 4c). For coverage, MS_C coverage was higher in the LA and HC groups than in the HA group (LA vs. HA: *t* = 3.238, *p* = 0.004, *Cohen’s d* = 1.270; HC vs. HA: *t* = 5.695, *p* < 0.001, *Cohen’s d* = 1.940), with no significant differences between the LA and the HC groups. MS_D coverage was lower in the HA group than in both the HC (*t* = 4.069, *p* < 0.001, *Cohen’s d* = 1.738) and LA groups (*t* = 2.272, *p* = 0.035, *Cohen’s d* = 0.694). No coverage differences were observed for MS_A and MS_B (Figure 4d). For TPs, no significant between‐group differences were found post‐intervention (Figure 4e–g–g).

To further assess whether the two anhedonia subtypes responded differentially to iTBS, a two‐way repeated‐measures ANOVA was performed with group (LA, HA) as between‐subject factor and time (pre, post) as within‐subject factor. Significant group × time interactions were observed for MS_B duration (*F*(*1*,*48*) = 15.983, *p* < 0.001, *η^2^* = 0.262), MS_C coverage (*F*(*1*,*48*) = 15.787, *p* < 0.001, *η^2^* = 0.397), MS_D occurrence (*F*(*1*,*48*) = 23.653, *p* < 0.001, *η^2^* = 0.496), and MS_D coverage (*F*(*1*,*48*) = 6.201, *p* = 0.021, *η^2^* = 0.228). Full ANOVA results are provided in Supporting Information 1: Table S3.

### 3.7. Correlation Between Clinical Improvement and Microstate Changes

Spearman correlation analyses were performed separately for the LA and HA groups. In the LA group, no significant correlations were observed between clinical improvement (ΔSHAPS, ΔHAMD‐17, and ΔHAMA‐14) and changes in microstate parameters (all *p* > 0.05). In the HA group, anhedonia improvement (ΔSHAPS) correlated positively with changes in MS_A duration (*r* = 0.622, *p* < 0.001) and negatively with changes in MS_B duration (*r* = −0.477, p = 0.016). Full correlation matrices are provided in Supporting Information 1: Table S5.

## 4. Discussion

Our study demonstrates distinct, subtype‐specific alterations in EEG microstate dynamics among MDD patients with anhedonia, alongside differential responsiveness to iTBS. At baseline, both the LA and the HA groups showed shortened MS_A duration, whereas the HA group additionally exhibited reductions in MS_C and MS_D, which have been associated with the DMN and DAN, respectively. The LA group, in contrast, showed selective abnormalities in MS_B duration and occurrence, suggesting early auditory–visual and reward‐related processing deficits. Across occurrence, coverage, and TP parameters, the HA group exhibited more pervasive disruptions, including globally diminished inter‐microstate transitions, suggesting large‐scale network integration deficits. Following iTBS, microstate parameters in the LA group recovered to levels comparable to HCs, whereas the HA group retained persistent deficits in MS_D dynamics and MS_C coverage. Correlation analyses further revealed that, within the HA group, greater improvement in anhedonia was associated with increased MS_A and decreased MS_B duration, linking clinical recovery to SN reorganization. Collectively, these findings provide novel evidence for subtype‐specific neural signatures of anhedonia and highlight the differential modulatory effects of iTBS on large‐scale brain networks. Notably, although iTBS produced antidepressant and anxiolytic effects in both subtypes, recovery of reward‐related functioning remained incomplete in the HA group, suggesting entrenched deficits in reward circuitry that may necessitate prolonged or multimodal interventions.

### 4.1. Specific Alterations of Anhedonia in EEG Microstates at Baseline

At baseline, both the LA and the HA groups showed shorter MS_A duration than HCs, suggesting reduced functional engagement of the AN. Source localization studies have consistently linked MS_A to the bilateral superior and middle temporal gyri, regions critical for auditory processing [51]. The observed reductions in MS_A duration align with altered sensory input processing and weakened auditory–emotional coupling, a pattern previously documented in depressive disorders [35, 52]. Beyond this shared deficit, the LA group showed reduced MS_B duration and occurrence relative to HCs, indicating hypoactivity in the VN and impaired integration of visual‐reward cues, consistent with the motivational dimension of anhedonia [53]. In contrast, the HA group exhibited shorter MS_C and MS_D durations than both the LA and HC groups. Source localization studies suggest that MS_C corresponds to regions within the DMN and MS_D to the DAN [54]—associations that, although derived from group‐level data, offer a useful interpretive framework [55]. These findings suggest that the HA group may have alterations in the temporal dynamics of networks supporting internally directed cognition and attentional regulation. Coverage and TP patterns further underscored these distinctions. Both patient groups showed reduced MS_A and MS_D coverage compared to HCs, whereas MS_C coverage was lower only in the HA group, consistent with more severe DMN inefficiency [53, 56]. Moreover, the HA group showed globally reduced TPs across nearly all state pairs, indicating pervasive disruption of whole‐brain coordination [57]. In contrast, the LA group exhibited TP patterns for specific state transitions (e.g., from MS_C to MS_B, from MS_C to MS_D) that were comparable to those of HCs, suggesting relatively selective alterations in dynamic switching mechanisms. Collectively, these baseline differences suggest that, while the LA group primarily shows selective sensory and perceptual network alterations, the HA group exhibits more widespread integration differences involving the AN, VN, DMN, and DAN.

### 4.2. Differential Microstate Changes Following iTBS Intervention

Following a 2‐week course of left DLPFC‐targeted iTBS, microstate parameters in the LA group no longer differed from those of HCs, suggesting possible changes in functional brain network dynamics. In contrast, the HA group exhibited only partial changes, with persistent abnormalities in several key parameters, indicative of reduced network plasticity. Post‐intervention, both groups showed increases in MS_A duration, occurrence, and coverage, reaching levels comparable to those of HCs. Given the established association between MS_A and AN activity [33], these changes may reflect enhanced auditory–emotional integration mediated by the superior and middle temporal gyri [51]. Clinically, this could facilitate improved emotional responsiveness to auditory cues, indirectly alleviating mood symptoms.

For MS_B, the LA group showed resolution of duration and occurrence abnormalities after treatment, consistent with enhanced VN engagement and improved visual‐reward pathway function [15]. However, the HA group’s MS_B duration remained significantly lower than that of the LA and HC groups, despite comparable occurrence and coverage. This persistent deficit may reflect structural or connectivity abnormalities between the VN and the DMN [58], compounded by dopaminergic dysfunction in the mesolimbic reward system [59], which may limit VN sensitivity to reward‐related visual inputs. Regarding MS_C, the HA group showed significant post‐treatment increases in duration and occurrence, such that these parameters no longer differed from HCs. However, coverage remained significantly lower than that of both the LA and the HC groups. Given that MS_C coverage reflects the proportion of recording time occupied by DMN‐related processing [53, 59], this persistent deficit may indicate sustained inefficiency in integrating multisensory and internally directed cognitive processes, potentially due to long‐standing pathology, although direct neurobiological measures are lacking [60].

For MS_D, the LA group showed no abnormalities post‐intervention, consistent with restored DAN‐mediated attentional control [61]. Conversely, the HA group retained significantly reduced MS_D duration, occurrence, and coverage compared with both the LA and HC groups. These findings align with prior reports in depression [62, 63] and point to enduring coordination deficits between the DAN and DMN [64, 65]. These DAN impairments may be particularly critical, as they likely underlie the HA group’s persistent difficulty in shifting attention toward rewarding stimuli.

### 4.3. Transition Probability Alterations and Network Switching Mechanisms

At baseline, TPs analyses revealed distinct profiles between the two anhedonia subtypes. The LA group exhibited selective baseline impairments but preserved connectivity in specific state pairs (e.g., from MS_B to MS_A, from MS_B to MS_C, and from MS_B to MS_D), suggesting mild disruption of SN–DMN coupling [57]. Following iTBS, these alterations resolved to levels comparable to those of HCs, suggesting improved dynamic switching efficiency. Mechanistically, this improvement may reflect enhanced transfer of sensory information into the DMN, enabling efficient engagement of pleasure‐processing pathways upon encountering rewarding stimuli [66].

In contrast, the HA group displayed globally reduced TPs across all microstate pairs at baseline, indicative of widespread dynamic coordination deficits involving the SN, DMN, and DAN [67, 68]. Post‐intervention, TPs no longer differed significantly between groups, indicating that iTBS enhanced dynamic switching efficiency in both subtypes. The convergence of post‐treatment TPs suggests that iTBS may promote global network coordination, potentially by optimizing sensory information transfer and cross‐network communication. However, the absence of residual TP differences between the HA and the HC groups should be interpreted cautiously. The HA group continued to show persistent abnormalities in temporal microstate parameters (e.g., MS_D coverage), indicating that TP normalization does not fully capture all aspects of network dysfunction.

### 4.4. Correlation Between Clinical Improvement and Microstate Changes

In the HA group, greater anhedonia improvement was associated with increased MS_A duration and decreased MS_B duration, whereas no significant correlations were observed in the LA group. This differential pattern further supports our core finding that the two anhedonia subtypes possess distinct neurophysiological profiles. The positive correlation between ΔSHAPS and ΔMS_A duration aligns with previous findings linking MS_A to avolition‐apathy symptoms, suggesting that AN engagement may be relevant to hedonic capacity [68]. Conversely, the negative correlation between ΔSHAPS and ΔMS_B duration is consistent with a recent study reporting that MS_B occurrence negatively correlates with baseline anhedonia severity in MDD [69]. The absence of correlations in the LA group may reflect a ceiling effect, as this group showed near‐complete resolution of microstate parameters, limiting variability and thus statistical power. These correlations should be interpreted cautiously due to the modest sample size, and future studies with larger cohorts are needed to confirm these subtype‐specific associations.

### 4.5. Clinical Implications and Limitations

These findings suggest that EEG microstate analysis may hold promise for subtype‐guided neuromodulation in MDD patients with anhedonia. Specifically, MS_D dynamics represent candidate electrophysiological signatures for treatment stratification: the LA group may respond adequately to standard iTBS protocols, whereas the HA group may require more intensive interventions targeting DAN‐reward circuit integration. Targeting the left DLPFC with iTBS appears sufficient to improve SN–DMN connectivity in the LA group, whereas additional network‐specific modulation—potentially via multisite stimulation or closed‐loop EEG‐guided protocols—may be necessary to address the more severe multinetwork integration deficits in the HA group.

Several limitations warrant consideration. First, the absence of a sham control group limits causal inference; without a placebo comparator, it is not possible to disentangle iTBS‐specific neuromodulatory effects from nonspecific influences such as expectancy effects and therapeutic alliance. Consequently, interpretations regarding treatment‐specific effects should be viewed cautiously. Second, although the observed effect sizes were large (*Cohen’s d* ranging from 0.6 to 4.3) and the sample size (*n* = 25 per group) was sufficient to detect significant group × time interactions, replication across larger, multicenter cohorts is needed to account for neurobiological heterogeneity among depressive subtypes and to validate the robustness of these findings. Third, the 2‐week intervention period may be insufficient to capture delayed network reorganization or sustained clinical effects; longitudinal follow‐up is essential to determine whether persistent abnormalities in the HA group reflect delayed recovery or true treatment resistance. Fourth, medication heterogeneity and reliance on a single instrument (SHAPS) for subtype classification constitute additional limitations. Regarding medication heterogeneity, sensitivity analyses excluding medication‐free patients yielded a pattern of results consistent with the main findings (Supporting information 2: Figure S1), suggesting that the observed effects were not solely driven by medication status. Additionally, the GEV values observed in the present study (~64%) are comparable to those reported in previous clinical MDD microstate studies. For example, Murphy et al. [35] reported GEV values of ~60% across HCs, current MDD patients, and remitted MDD patients. Importantly, in the present study, no significant GEV differences were observed among groups or across sessions, suggesting that the between‐group and longitudinal comparisons were unlikely to be driven by differences in the explanatory power of the microstate model. Nevertheless, future studies should incorporate detailed medication records and employ stratified analyses or covariate adjustments to further isolate treatment effects from pharmacological influences. Regarding subtype classification, future studies should incorporate multimodal assessments (e.g., clinical interviews, reward‐related behavioral tasks) or data‐driven clustering approaches to further validate subtype classification. Finally, while our findings suggest that persistent anhedonia in the HA group is linked to deficits in visual‐reward and attention integration, the precise neurochemical mechanisms remain unclear. Combining EEG microstate analysis with multimodal imaging (e.g., fMRI, PET) could provide converging evidence for the structural and neurotransmitter‐level underpinnings of these alterations.

## 5. Conclusions

This study used EEG microstate analysis to clarify subtype‐specific neural alterations of anhedonia in MDD and their responsiveness to iTBS. We found that microstate parameters in the LA group showed resolution of alterations after treatment to levels comparable to those of HCs, whereas the HA group exhibited only partial changes, with persistent abnormalities in networks associated with the SN, DMN, and DAN. Notably, in the HA group, greater clinical improvement was associated with increased MS_A duration and decreased MS_B duration, suggesting that symptom recovery in this subtype is specifically coupled with SN reorganization. These findings indicate that anhedonia subtypes are underpinned by distinct large‐scale network dysfunctions that differentially shape treatment outcomes. Importantly, EEG microstate features, particularly MS_D dynamics, may represent candidate electrophysiological signatures for patient stratification and may inform the development of subtype‐guided neuromodulation strategies targeting refractory anhedonia in MDD.

## Author Contributions

Jiang Wu designed the study and wrote the first draft of the manuscript. Zhengzhi Deng, Xinyuan Cheng, and Lan Hu recruited the sample. Li Pu finished the clinical assessments. Shaoqing Li, Zhihong Chen, and Nan Qiu conducted the statistical analyses. Hongmei Yan and Dezhong Yao revised the manuscript and supervised the project.

## Funding

This work was supported by the Brain Science and Brain‐like Intelligence Technology‐ National Science and Technology Major Project (Grant 2022ZD0208500), the National Natural Science Foundation of China (Grant 62276051), the S&T Special Program of Huzhou (Grant 2024GZ05), and the Chengdu Municipal Health Commission (Grant 2022637).

## Disclosure

All authors contributed to and have approved the final manuscript.

## Ethics Statement

This study was approved by the institutional review board of the Ethics Committee of Chengdu Fourth People’s Hospital (Sichuan Province, China). All methods were carried out in accordance with relevant guidelines and regulations.

## Conflicts of Interest

The authors declare no conflicts of interest.

## Supporting Information

Additional supporting information can be found online in the Supporting Information section.

## Supporting information


**Supporting Information 1** Table S1 summarizes the clinical characteristics and comparability of the LA HA groups. Table S2 presents the complete results of all transition probability comparisons, including raw and FDR‐corrected *p*‐values, across all group pairs and time points. Table S3 reports the full repeated‐measures ANOVA results for all microstate parameters, including group × time interaction effects and effect sizes. Table S4 lists the number of artifact‐free EEG epochs for each participant, confirming data quality across sessions. Table S5 provides the complete correlation matrices between clinical improvements and changes in microstate parameters for both patient groups. Table S6 presents GEV statistics, demonstrating that the four‐class microstate model provided comparable explanatory power across groups and time points.

**Supporting Information 2** Figure S1 shows the results of a sensitivity analysis excluding medication‐free patients, confirming that the main findings were not driven by medication heterogeneity.

## Data Availability

The data in the current study are available from the corresponding author upon reasonable request.
