# Supplementary material for: EEG Microstate Alterations in Anhedonia Subtypes of Major Depressive Disorder and Their Modulation by Intermittent Theta‐Burst Stimulation
Source: Depress Anxiety. 2026 Sep 21;2026:1982576. doi: 10.1155/da/1982576 (PMC13591540; doi:10.1155/da/1982576)
Supplement: Supplementary file 1 [file DA-2026-1982576-s001.pdf]

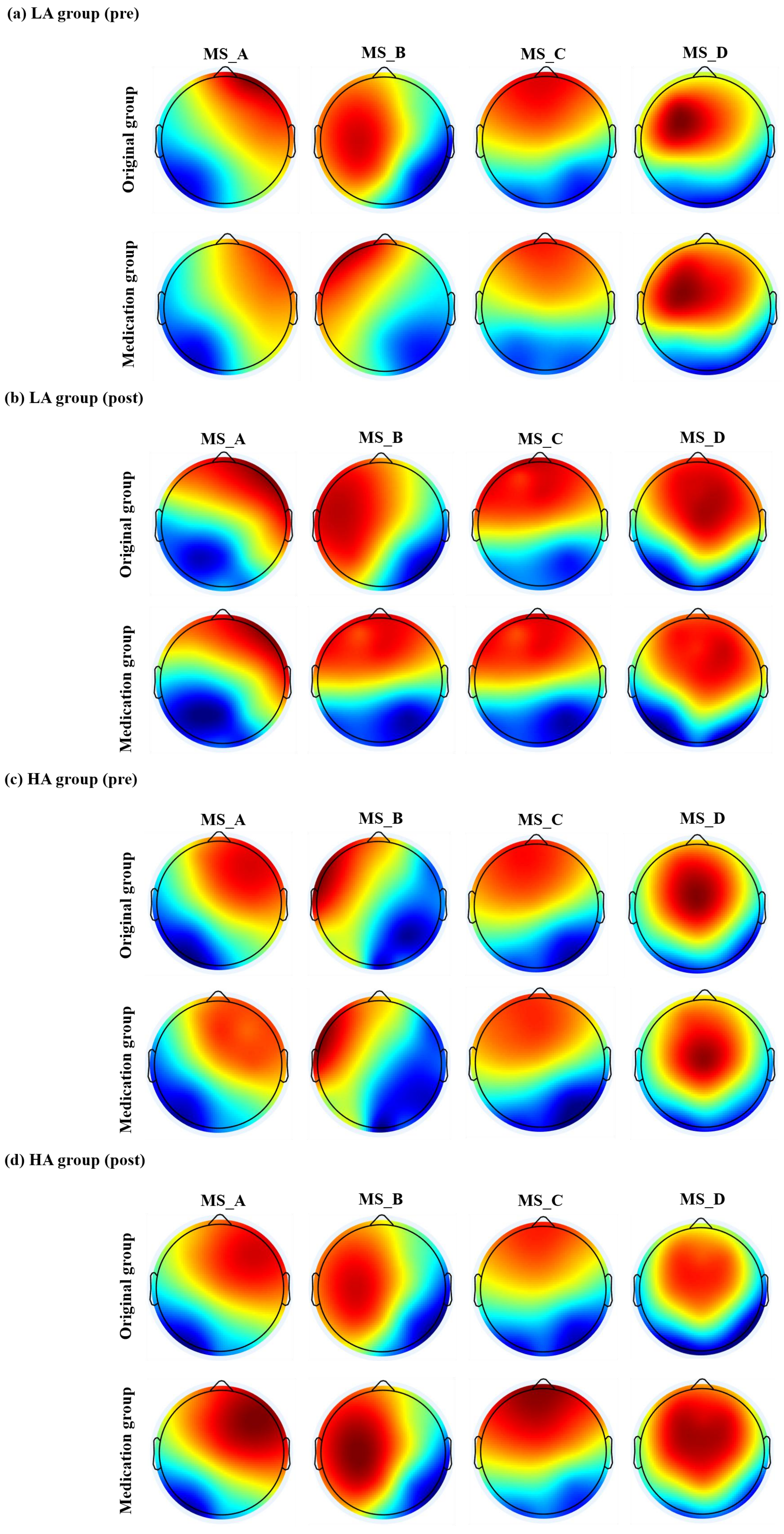
(a) LA group (pre)
MS_A
MS_B
MS_C
MS_D
Original group
Medication group
(b) LA group (post)
MS_A
MS_B
MS_C
MS_D
Original group
Medication group
(c) HA group (pre)
MS_A
MS_B
MS_C
MS_D
Original group
Medication group
(d) HA group (post)
MS_A
MS_B
MS_C
MS_D
Original group
Medication group
