# Supplementary material for: EEG Microstate Alterations in Anhedonia Subtypes of Major Depressive Disorder and Their Modulation by Intermittent Theta‐Burst Stimulation
Source: Depress Anxiety. 2026 Sep 21;2026:1982576. doi: 10.1155/da/1982576 (PMC13591540; doi:10.1155/da/1982576)
Supplement: Supplementary file 2 [file DA-2026-1982576-s002.doc]

**Supplementary Materials**

**S1. Clinical characteristics and comparability of LA and HA groups**

**Table S1 Clinical Characteristics of LA and HA groups.**

| **Characteristic** | **LA group** | **HA group** | **Test statistic** | ***p*** | ***η²*** |
| --- | --- | --- | --- | --- | --- |
| Illness duration (months), median (IQR) | 5.0  (1.0–36.0) | 12.0  (1.5-24.0) | U = 307.00 | 0.846 | -- |
| First-episode, n (%) | 15 (60.00%) | 14 (56.00%) | χ² = 0.0802 | 0.774 | 0.002 |
| Medication status, n (%) | | | | |  |
| Medication-free during study | 5 (20.00%) | 5 (20.00%) | χ² = 0.010 | 0.920 | 0.0002 |
| Stable SSRI/SNRI during study | 20 (80.00%) | 20 (80.00%) |  |  |  |

Note: Illness duration was non-normally distributed; thus, median and Mann-Whitney U test are reported. All medicated patients (n = 20 per group) had been receiving stable doses of SSRIs or SNRIs for at least 3 months prior to enrollment and continued their regimens throughout the study period. Abbreviations: LA, low anhedonia; HA, high anhedonia; IQR, interquartile range; SSRI, selective serotonin reuptake inhibitor; SNRI, serotonin-norepinephrine reuptake inhibitor; χ², chi-square test.

**S2. Full Transition Probability Statistics Across All Comparison Families**

Table S2. Complete results of transition probability comparisons.

| Comparison | Transition | *t* | *df* | *p*  (Raw ) | *p*  (FDR_corrected) | *Cohen's d* |
| --- | --- | --- | --- | --- | --- | --- |
| HC group  *vs.*  LA group (*pre*) | A to B | 2.193 | 48 | 0.033 | > 0.05 | 0.633 |
|  | A to C | 2.243 | 48 | 0.030 | > 0.05 | 0.648 |
|  | A to D | 2.291 | 48 | 0.027 | > 0.05 | 0.662 |
|  | `B to A | 3.027 | 48 | 0.004 | 0.020 | 0.874 |
|  | B to C | 3.989 | 48 | < 0.001 | 0.004 | 1.152 |
|  | B to D | 2.655 | 48 | 0.011 | 0.037 | 0.767 |
|  | C to A | 1.708 | 48 | 0.094 | > 0.05 | 0.483 |
|  | C to B | 1.793 | 48 | 0.079 | > 0.05 | 0.507 |
|  | C to D | 0.599 | 48 | 0.552 | > 0.05 | 0.169 |
|  | D to A | 2.103 | 48 | 0.041 | > 0.05 | 0.607 |
|  | D to B | 0.336 | 48 | 0.738 | > 0.05 | 0.095 |
|  | D to C | 0.841 | 48 | 0.405 | > 0.05 | 0.238 |
| HC group  *vs.*  HA group (*pre*) | A to B | 2.832 | 48 | 0.007 | 0.020 | 0.801 |
|  | A to C | 2.736 | 48 | 0.009 | 0.013 | 0.774 |
|  | A to D | 2.706 | 48 | 0.009 | 0.012 | 0.765 |
|  | B to A | 3.066 | 48 | 0.004 | 0.043 | 0.867 |
|  | B to C | 2.808 | 48 | 0.007 | 0.017 | 0.794 |
|  | B to D | 2.877 | 48 | 0.006 | 0.024 | 0.814 |
|  | C to A | 2.768 | 48 | 0.008 | 0.016 | 0.783 |
|  | C to B | 2.927 | 48 | 0.005 | 0.031 | 0.828 |
|  | C to D | 2.584 | 48 | 0.129 | 0.014 | 0.731 |
|  | D to A | 2.744 | 48 | 0.009 | 0.015 | 0.776 |
|  | D to B | 2.656 | 48 | 0.011 | 0.013 | 0.751 |
|  | D to C | 2.467 | 48 | 0.017 | 0.017 | 0.698 |
| LA group (*pre*)  *vs.*  HA group (*pre*) | A to B | 0.812 | 48 | 0.421 | > 0.05 | 0.230 |
|  | A to C | 0.609 | 48 | 0.546 | > 0.05 | 0.172 |
|  | A to D | 0.611 | 48 | 0.544 | > 0.05 | 0.173 |
|  | B to A | -0.065 | 48 | 0.948 | > 0.05 | -0.019 |
|  | B to C | -3.235 | 48 | 0.002 | 0.018 | -0.915 |
|  | B to D | 0.261 | 48 | 0.795 | > 0.05 | 0.074 |
|  | C to A | 1.524 | 48 | 0.134 | > 0.05 | 0.431 |
|  | C to B | 1.435 | 48 | 0.158 | > 0.05 | 0.406 |
|  | C to D | 2.740 | 48 | 0.009 | 0.026 | 0.825 |
|  | D to A | 0.839 | 48 | 0.406 | > 0.05 | 0.237 |
|  | D to B | 3.389 | 48 | 0.002 | 0.009 | 1.020 |
|  | D to C | 2.118 | 48 | 0.039 | > 0.05 | 0.599 |
| HC group  *vs.*  LA group (*post*) | A to B | 1.431 | 48 | 0.159 | > 0.05 | 0.405 |
|  | A to C | 0.153 | 48 | 0.879 | > 0.05 | 0.043 |
|  | A to D | 1.759 | 48 | 0.085 | > 0.05 | 0.498 |
|  | B to A | 1.821 | 48 | 0.075 | > 0.05 | 0.515 |
|  | B to C | 0.475 | 48 | 0.636 | > 0.05 | 0.134 |
|  | B to D | 1.942 | 48 | 0.058 | > 0.05 | 0.549 |
|  | C to A | 0.061 | 48 | 0.952 | > 0.05 | 0.017 |
|  | C to B | 0.387 | 48 | 0.701 | > 0.05 | 0.109 |
|  | C to D | 1.254 | 48 | 0.216 | > 0.05 | 0.355 |
|  | D to A | 1.940 | 48 | 0.058 | > 0.05 | 0.549 |
|  | D to B | 1.936 | 48 | 0.059 | > 0.05 | 0.548 |
|  | D to C | 1.316 | 48 | 0.194 | > 0.05 | 0.372 |
| HC group  *vs.*  HA group (*post*) | A to B | 1.387 | 48 | 0.172 | > 0.05 | 0.392 |
|  | A to C | 0.948 | 48 | 0.348 | > 0.05 | 0.268 |
|  | A to D | 1.992 | 48 | 0.052 | > 0.05 | 0.563 |
|  | B to A | 1.804 | 48 | 0.077 | > 0.05 | 0.510 |
|  | B to C | 1.176 | 48 | 0.246 | > 0.05 | 0.332 |
|  | B to D | 2.543 | 48 | 0.014 | > 0.05 | 0.751 |
|  | C to A | 0.992 | 48 | 0.326 | > 0.05 | 0.281 |
|  | C to B | 1.141 | 48 | 0.259 | > 0.05 | 0.323 |
|  | C to D | 1.907 | 48 | 0.063 | > 0.05 | 0.540 |
|  | D to A | 2.182 | 48 | 0.034 | > 0.05 | 0.644 |
|  | D to B | 2.464 | 48 | 0.018 | > 0.05 | 0.727 |
|  | D to C | 1.981 | 48 | 0.054 | > 0.05 | 0.560 |
| LA group (*post*)  *vs.*  HA group (*post*) | A to B | 0.095 | 48 | 0.925 | > 0.05 | 0.027 |
|  | A to C | 0.815 | 48 | 0.429 | > 0.05 | 0.230 |
|  | A to D | 0.297 | 48 | 0.768 | > 0.05 | 0.084 |
|  | B to A | 0.132 | 48 | 0.896 | > 0.05 | 0.037 |
|  | B to C | 0.762 | 48 | 0.450 | > 0.05 | 0.216 |
|  | B to D | 0.636 | 48 | 0.528 | > 0.05 | 0.180 |
|  | C to A | 0.919 | 48 | 0.363 | > 0.05 | 0.260 |
|  | C to B | 0.773 | 48 | 0.443 | > 0.05 | 0.219 |
|  | C to D | 0.914 | 48 | 0.365 | > 0.05 | 0.259 |
|  | D to A | 0.398 | 48 | 0.692 | > 0.05 | 0.113 |
|  | D to B | 0.650 | 48 | 0.519 | > 0.05 | 0.184 |
|  | D to C | 0.952 | 48 | 0.346 | > 0.05 | 0.269 |
| LA group  (*pre vs. post*) | A to B | 0.970 | 24 | 0.342 | > 0.05 | 0.194 |
|  | A to C | 2.086 | 24 | 0.043 | > 0.05 | 0.427 |
|  | A to D | 0.568 | 24 | 0.576 | > 0.05 | 0.114 |
|  | B to A | 1.784 | 24 | 0.087 | > 0.05 | 0.357 |
|  | B to C | 3.760 | 24 | 0.001 | 0.016 | 0.784 |
|  | B to D | 0.628 | 24 | 0.536 | > 0.05 | 0.126 |
|  | C to A | 1.542 | 24 | 0.136 | > 0.05 | 0.308 |
|  | C to B | 1.485 | 24 | 0.151 | > 0.05 | 0.297 |
|  | C to D | -0.924 | 24 | 0.365 | > 0.05 | -0.185 |
|  | D to A | 0.143 | 24 | 0.888 | > 0.05 | 0.029 |
|  | D to B | -2.089 | 24 | 0.048 | > 0.05 | -0.418 |
|  | D to C | -0.597 | 24 | 0.556 | > 0.05 | -0.119 |
| HA group  (*pre vs. post*) | A to B | 1.378 | 24 | 0.181 | > 0.05 | 0.276 |
|  | A to C | 1.516 | 24 | 0.143 | > 0.05 | 0.303 |
|  | A to D | 0.649 | 24 | 0.523 | > 0.05 | 0.130 |
|  | B to A | 1.358 | 24 | 0.187 | > 0.05 | 0.272 |
|  | B to C | 1.665 | 24 | 0.109 | > 0.05 | 0.333 |
|  | B to D | 0.261 | 24 | 0.796 | > 0.05 | 0.052 |
|  | C to A | 1.539 | 24 | 0.137 | > 0.05 | 0.308 |
|  | C to B | 1.684 | 24 | 0.105 | > 0.05 | 0.333 |
|  | C to D | 0.792 | 24 | 0.436 | > 0.05 | 0.158 |
|  | D to A | 0.435 | 24 | 0.668 | > 0.05 | 0.087 |
|  | D to B | 0.084 | 24 | 0.934 | > 0.05 | 0.017 |
|  | D to C | 0.600 | 24 | 0.554 | > 0.05 | 0.120 |

Notes: FDR correction (Benjamini-Hochberg) was applied separately to each family of comparisons: baseline group differences (36 comparisons), post-intervention group differences (36 comparisons), and pre-post changes (24 comparisons). For all comparisons, q = 0.05 was used. Significant results after FDR correction are indicated in the last column. Abbreviations: df, degrees of freedom; FDR, False Discovery Rate.

**S3. Repeated-measures ANOVA results for microstate parameters.**

Table S3. Repeated-measures ANOVA results for microstate parameters: Group × Time interactions and effect sizes

| **Microstate** | **Parameter** | **Effect** | ***F(1,48)*** | ***p*** | ***η²*** |
| --- | --- | --- | --- | --- | --- |
| MS_A | Duration | Time | 0.156 | 0.695 | 0.003 |
|  |  | Group | 0.084 | 0.774 | 0.002 |
|  |  | Interaction | 0.072 | 0.789 | 0.002 |
|  | Occurrence | Time | 3.622 | 0.068 | 0.122 |
|  |  | Group | 0.239 | 0.629 | 0.009 |
|  |  | Interaction | 3.383 | 0.077 | 0.115 |
|  | Coverage | Time | 0.136 | 0.716 | 0.006 |
|  |  | Group | 0.132 | 0.720 | 0.006 |
|  |  | Interaction | 0.004 | 0.952 | 0.000 |
| MS_B | Duration | Time | 17.087 | < 0.001 | 0.275 |
|  |  | Group | 1.104 | 0.299 | 0.024 |
|  |  | Interaction | 15.983 | <0.001 | 0.262 |
|  | Occurrence | Time | 3.402 | 0.076 | 0.108 |
|  |  | Group | 2.433 | 0.130 | 0.08 |
|  |  | Interaction | 0.969 | 0.333 | 0.033 |
|  | Coverage | Time | 0.015 | 0.903 | 0.001 |
|  |  | Group | 0.000 | 0.987 | 0.000 |
|  |  | Interaction | 0.015 | 0.904 | 0.001 |
| MS_C | Duration | Time | 3.697 | 0.061 | 0.076 |
|  |  | Group | 3.158 | 0.082 | 0.066 |
|  |  | Interaction | 0.540 | 0.466 | 0.012 |
|  | Occurrence | Time | 7.213 | 0.012 | 0.205 |
|  |  | Group | 5.385 | 0.028 | 0.161 |
|  |  | Interaction | 1.828 | 0.187 | 0.061 |
|  | Coverage | Time | 15.790 | < 0.001 | 0.397 |
|  |  | Group | 15.787 | < 0.001 | 0.397 |
|  |  | Interaction | 0.003 | 0.957 | 0.000 |
| MS_D | Duration | Time | 17.480 | < 0.001 | 0.280 |
|  |  | Group | 17.383 | < 0.001 | 0.279 |
|  |  | Interaction | 0.096 | 0.758 | 0.002 |
|  | Occurrence | Time | 32.945 | < 0.001 | 0.578 |
|  |  | Group | 9.292 | 0.006 | 0.279 |
|  |  | Interaction | 23.653 | < 0.001 | 0.496 |
|  | Coverage | Time | 9.197 | 0.098 | 0.125 |
|  |  | Group | 2.996 | 0.098 | 0.125 |
|  |  | Interaction | 6.201 | 0.0212 | 0.228 |

Note: Two-way repeated-measures ANOVA was performed with Group (LA, HA) as between-subject factor and Time (*pre, post*) as within-subject factor for each microstate parameter. Degrees of freedom are (1, 48) for all analyses unless otherwise indicated. Abbreviations: *η²*, partial eta squared (effect size).

Table S4. Number of artifact-free epochs for each participant in the LA and HA groups across Pre- and Post-intervention sessions.

| **Subject_ID** | **LA group (*pre*)** | **LA group (*post*)** | **HA group (*pre*)** | **HA group (*post*)** |
| --- | --- | --- | --- | --- |
| 001 | 120 | 100 | 120 | 100 |
| 002 | 106 | 120 | 120 | 115 |
| 003 | 116 | 120 | 119 | 102 |
| 004 | 101 | 108 | 107 | 120 |
| 005 | 110 | 120 | 120 | 101 |
| 006 | 111 | 100 | 116 | 102 |
| 007 | 120 | 120 | 115 | 116 |
| 008 | 111 | 100 | 116 | 119 |
| 009 | 120 | 114 | 115 | 120 |
| 010 | 119 | 101 | 108 | 120 |
| 011 | 119 | 100 | 120 | 105 |
| 012 | 118 | 120 | 119 | 116 |
| 013 | 119 | 119 | 115 | 120 |
| 014 | 101 | 119 | 120 | 118 |
| 015 | 120 | 119 | 105 | 119 |
| 016 | 101 | 101 | 116 | 120 |
| 017 | 107 | 120 | 120 | 102 |
| 018 | 120 | 115 | 118 | 112 |
| 019 | 120 | 107 | 119 | 114 |
| 020 | 101 | 101 | 116 | 109 |
| 021 | 102 | 108 | 110 | 100 |
| 022 | 115 | 120 | 100 | 120 |
| 023 | 100 | 101 | 108 | 120 |
| 024 | 100 | 114 | 115 | 118 |
| 025 | 112 | 120 | 119 | 100 |
| **Mean ± SD** | 111.72 ± 8.19 | 111.48 ± 8.63 | 115.04 ± 5.56 | 112.32 ± 8.09 |

**S4. Correlation analysis.**

**Table S5. Correlations between clinical improvement and changes in EEG microstate parameters in LA and HA groups.**

| **Group** | **Clinical measure** | **Microstate parameter** | ***r*** | ***p*** |
| --- | --- | --- | --- | --- |
| **LA group** | ΔSHAPS | ΔMS_A duration | -0.068 | 0.748 |
|  |  | ΔMS_B duration | -0.193 | 0.356 |
|  |  | ΔMS_C duration | -0.269 | 0.192 |
|  |  | ΔMS_D duration | -0.137 | 0.514 |
|  |  | ΔMS_A coverage | 0.011 | 0.958 |
|  |  | ΔMS_B coverage | 0.095 | 0.649 |
|  |  | ΔMS_C coverage | 0.075 | 0.723 |
|  |  | ΔMS_D coverage | -0.196 | 0.348 |
|  |  | ΔMS_A occurrence | -0.134 | 0.524 |
|  |  | ΔMS_B occurrence | 0.045 | 0.829 |
|  |  | ΔMS_C occurrence | -0.126 | 0.546 |
|  |  | ΔMS_D occurrence | 0.056 | 0.792 |
|  |  | ΔTP A to B | -0.053 | 0.799 |
|  |  | ΔTP A to C | -0.069 | 0.738 |
|  |  | ΔTP A to D | -0.089 | 0.669 |
|  |  | ΔTP `B to A | 0.005 | 0.982 |
|  |  | ΔTP B to C | 0.005 | 0.983 |
|  |  | ΔTP B to D | 0.138 | 0.509 |
|  |  | ΔTP C to A | -0.059 | 0.777 |
|  |  | ΔTP C to B | 0.137 | 0.513 |
|  |  | ΔTP C to D | 0.105 | 0.616 |
|  |  | ΔTP D to A | 0.052 | 0.807 |
|  |  | ΔTP D to B | 0.122 | 0.563 |
|  |  | ΔTP D to C | 0.072 | 0.729 |
|  | ΔHAMD | ΔMS_A duration | -0.067 | 0.747 |
|  |  | ΔMS_B duration | 0.046 | 0.826 |
|  |  | ΔMS_C duration | 0.101 | 0.634 |
|  |  | ΔMS_D duration | -0.082 | 0.695 |
|  |  | ΔMS_A coverage | -0.142 | 0.449 |
|  |  | ΔMS_B coverage | -0.360 | 0.077 |
|  |  | ΔMS_C coverage | 0.010 | 0.963 |
|  |  | ΔMS_D coverage | -0.029 | 0.891 |
|  |  | ΔMS_A occurrence | 0.276 | 0.180 |
|  |  | ΔMS_B occurrence | 0.076 | 0.719 |
|  |  | ΔMS_C occurrence | 0.158 | 0.452 |
|  |  | ΔMS_D occurrence | -0.028 | 0.896 |
|  |  | ΔTP A to B | 0.041 | 0.847 |
|  |  | ΔTP A to C | 0.161 | 0.442 |
|  |  | ΔTP A to D | -0.036 | 0.866 |
|  |  | ΔTP `B to A | 0.094 | 0.643 |
|  |  | ΔTP B to C | 0.201 | 0.335 |
|  |  | ΔTP B to D | 0.029 | 0.889 |
|  |  | ΔTP C to A | 0.096 | 0.647 |
|  |  | ΔTP C to B | 0.109 | 0.602 |
|  |  | ΔTP C to D | -0.087 | 0.680 |
|  |  | ΔTP D to A | -0.014 | 0.946 |
|  |  | ΔTP D to B | 0.099 | 0.637 |
|  |  | ΔTP D to C | 0.031 | 0.886 |
|  | ΔHAMA | ΔMS_A duration | -0.126 | 0.549 |
|  |  | ΔMS_B duration | -0.155 | 0.461 |
|  |  | ΔMS_C duration | -0.202 | 0.334 |
|  |  | ΔMS_D duration | -0.138 | 0.510 |
|  |  | ΔMS_A coverage | -0.344 | 0.177 |
|  |  | ΔMS_B coverage | 0.024 | 0.910 |
|  |  | ΔMS_C coverage | 0.051 | 0.811 |
|  |  | ΔMS_D coverage | -0.153 | 0.449 |
|  |  | ΔMS_A occurrence | 0.008 | 0.948 |
|  |  | ΔMS_B occurrence | 0.114 | 0.587 |
|  |  | ΔMS_C occurrence | 0.219 | 0.293 |
|  |  | ΔMS_D occurrence | 0.024 | 0.908 |
|  |  | ΔTP A to B | -0.053 | 0.799 |
|  |  | ΔTP A to C | -0.067 | 0.739 |
|  |  | ΔTP A to D | -0.089 | 0.669 |
|  |  | ΔTP `B to A | 0.005 | 0.982 |
|  |  | ΔTP B to C | 0.005 | 0.982 |
|  |  | ΔTP B to D | 0.139 | 0.508 |
|  |  | ΔTP C to A | -0.059 | 0.777 |
|  |  | ΔTP C to B | 0.137 | 0.512 |
|  |  | ΔTP C to D | 0.105 | 0.616 |
|  |  | ΔTP D to A | 0.052 | 0.807 |
|  |  | ΔTP D to B | 0.121 | 0.563 |
|  |  | ΔTP D to C | 0.072 | 0.729 |
| **HA group** | ΔSHAPS | ΔMS_A duration | 0.622 | < 0.001 |
|  |  | ΔMS_B duration | -0.477 | 0.016 |
|  |  | ΔMS_C duration | 0.215 | 0.917 |
|  |  | ΔMS_D duration | -0.084 | 0.689 |
|  |  | ΔMS_A coverage | 0.133 | 0.526 |
|  |  | ΔMS_B coverage | -0.246 | 0.237 |
|  |  | ΔMS_C coverage | -0.127 | 0.546 |
|  |  | ΔMS_D coverage | 0.153 | 0.465 |
|  |  | ΔMS_A occurrence | -0.049 | 0.816 |
|  |  | ΔMS_B occurrence | 0.211 | 0.312 |
|  |  | ΔMS_C occurrence | 0.286 | 0.165 |
|  |  | ΔMS_D occurrence | -0.167 | 0.425 |
|  |  | ΔTP A to B | -0.239 | 0.249 |
|  |  | ΔTP A to C | -0.233 | 0.262 |
|  |  | ΔTP A to D | -0.162 | 0.439 |
|  |  | ΔTP `B to A | -0.241 | 0.245 |
|  |  | ΔTP B to C | -0.297 | 0.149 |
|  |  | ΔTP B to D | -0.174 | 0.405 |
|  |  | ΔTP C to A | -0.234 | 0.259 |
|  |  | ΔTP C to B | -0.301 | 0.145 |
|  |  | ΔTP C to D | -0.086 | 0.685 |
|  |  | ΔTP D to A | -0.144 | 0.492 |
|  |  | ΔTP D to B | -0.158 | 0.452 |
|  |  | ΔTP D to C | -0.061 | 0.773 |
|  | ΔHAMD | ΔMS_A duration | 0.274 | 0.186 |
|  |  | ΔMS_B duration | -0.217 | 0.297 |
|  |  | ΔMS_C duration | -0.049 | 0.815 |
|  |  | ΔMS_D duration | 0.082 | 0.697 |
|  |  | ΔMS_A coverage | 0.290 | 0.159 |
|  |  | ΔMS_B coverage | -0.215 | 0.302 |
|  |  | ΔMS_C coverage | -0.144 | 0.494 |
|  |  | ΔMS_D coverage | -0.068 | 0.746 |
|  |  | ΔMS_A occurrence | 0.094 | 0.656 |
|  |  | ΔMS_B occurrence | -0.081 | 0.699 |
|  |  | ΔMS_C occurrence | 0.308 | 0.121 |
|  |  | ΔMS_D occurrence | -0.239 | 0.250 |
|  |  | ΔTP A to B | 0.006 | 0.977 |
|  |  | ΔTP A to C | -0.045 | 0.832 |
|  |  | ΔTP A to D | 0.018 | 0.934 |
|  |  | ΔTP `B to A | 0.007 | 0.974 |
|  |  | ΔTP B to C | -0.081 | 0.699 |
|  |  | ΔTP B to D | 0.107 | 0.609 |
|  |  | ΔTP C to A | -0.048 | 0.821 |
|  |  | ΔTP C to B | -0.077 | 0.716 |
|  |  | ΔTP C to D | -0.080 | 0.703 |
|  |  | ΔTP D to A | 0.027 | 0.898 |
|  |  | ΔTP D to B | 0.119 | 0.569 |
|  |  | ΔTP D to C | -0.086 | 0.683 |
|  | ΔHAMA | ΔMS_A duration | 0.063 | 0.511 |
|  |  | ΔMS_B duration | -0.119 | 0.811 |
|  |  | ΔMS_C duration | 0.050 | 0.571 |
|  |  | ΔMS_D duration | -0.138 | 0.745 |
|  |  | ΔMS_A coverage | -0.102 | 0.629 |
|  |  | ΔMS_B coverage | -0.218 | 0.296 |
|  |  | ΔMS_C coverage | -0.109 | 0.605 |
|  |  | ΔMS_D coverage | 0.069 | 0.745 |
|  |  | ΔMS_A occurrence | 0.244 | 0.239 |
|  |  | ΔMS_B occurrence | 0.345 | 0.091 |
|  |  | ΔMS_C occurrence | 0.357 | 0.080 |
|  |  | ΔMS_D occurrence | -0.146 | 0.486 |
|  |  | ΔTP A to B | -0.205 | 0.326 |
|  |  | ΔTP A to C | -0.212 | 0.309 |
|  |  | ΔTP A to D | -0.162 | 0.438 |
|  |  | ΔTP `B to A | -0.216 | 0.299 |
|  |  | ΔTP B to C | -0.269 | 0.192 |
|  |  | ΔTP B to D | -0.128 | 0.544 |
|  |  | ΔTP C to A | -0.220 | 0.290 |
|  |  | ΔTP C to B | -0.263 | 0.204 |
|  |  | ΔTP C to D | -0.200 | 0.338 |
|  |  | ΔTP D to A | -0.162 | 0.440 |
|  |  | ΔTP D to B | -0.112 | 0.594 |
|  |  | ΔTP D to C | -0.201 | 0.335 |

**S5. Sensitivity analysis excluding medication-free patients**

**Fig. S1**. Sensitivity analysis excluding medication-free patients. Microstate topographies (MS_A–MS_D) after excluding medication-free patients from both patient groups, retaining only those on stable SSRI/SNRI regimens (n = 20 per group for LA and HA; HC group unchanged, n = 25). (a) LA group at baseline; (b) LA group post-intervention; (c) HA group at baseline; (d) HA group post-intervention. The spatial configuration of all four microstates remained consistent with the primary analysis using the full sample (Fig. 1a, 2a, 3a, 4a), indicating that medication heterogeneity did not drive the observed microstate patterns. Color scaling represents normalized amplitude; polarity was ignored during clustering, consistent with the primary analysis.

**S6. Global explained variance (GEV) statistics**

To assess whether the four-class microstate model provided comparable explanatory power across groups and time points, we computed the global explained variance (GEV) for each participant at baseline and post-intervention. GEV was defined as the proportion of the total EEG signal variance accounted for by the fitted microstate model. One-way ANOVA was used to compare GEV among groups at baseline and post-intervention, and paired-samples t-tests were used to examine pre- to post-intervention changes within each patient group. The descriptive statistics and group comparison results are summarized in Table S6.

Table S6. Global explained variance (GEV) across groups and time points: descriptive statistics and statistical comparisons.

| **Group** | **Mean±SD (%)**  **(n = 25)** | **Comparison** | **Test** | ***F(2,72)* / *t*** | ***p*** | ***η²*** |
| --- | --- | --- | --- | --- | --- | --- |
| HC | 63.56 ± 8.39 | pre: HC *vs.* LA *vs.* HA | One-way ANOVA | 0.228 | 0.797 | 0.006 |
| LA group (pre) | 64.96 ± 8.14 | post: HC *vs.* LA_post *vs.* HA |  | 1.365 | 0.262 | 0.037 |
| LA group (post) | 63.08 ± 8.59 | LA: pre *vs.* post | Paired t-test | 0.891 | 0.382 | -- |
| HA group (pre) | 63.64 ± 8.17 | HA: pre *vs.* post |  | 1.358 | 0.187 | -- |
| HA group (post) | 67.16 ± 11.37 |  |  |  |  |  |

Note: HC, healthy control; LA, low anhedonia; HA, high anhedonia; SD, standard deviation; GEV, global explained variance. GEV was computed as the proportion of total EEG signal variance accounted for by the fitted four-class microstate model. No significant differences were observed among groups or across sessions, suggesting that the explanatory power of the microstate model was comparable across groups and remained stable over time. Because healthy controls did not receive an intervention, their baseline GEV values were used as the reference for post-intervention group comparisons.
